# Immune-related adverse events: A bibliometric analysis

**DOI:** 10.3389/fimmu.2022.1096806

**Published:** 2022-12-15

**Authors:** Shi-Tao Jiang, Yao-Ge Liu, Lei Zhang, Xin-Ting Sang, Yi-Yao Xu, Xin Lu

**Affiliations:** Department of Liver Surgery, State Key Laboratory of Complex Severe and Rare Diseases, Peking Union Medical College Hospital, Chinese Academy of Medical Sciences and Peking Union Medical College, Beijing, China

**Keywords:** immune-related adverse events, bibliometrics, VOSviewer, citespace, frontiers

## Abstract

**Background:**

Despite providing clinical benefit, immune checkpoint inhibitors (ICIs) can cause immune-related adverse events (irAEs) in a number of patients. This study explored the development pattern in irAEs research from a bibliometric perspective.

**Methods:**

We obtained articles and reviews related to irAEs from the Web of Science Core Collection (WoSCC) (retrieved on September 13, 2022). Using the R package “Bibliometrix”, the main bibliometric features were calculated, and a three-filed plot was generated to show the relationship between authors, institutions, and topics. VOSviewer was used for co-authorship and keyword co-occurrence analysis and visualization. CiteSpace was used to detect burst references and keywords.

**Results:**

A total of 3995 publications on irAEs were included. The United States (US), Japan, and China had the highest publications. The Journal for ImmunoTherapy of Cancer had the highest number of publications. In addition to “immune-related adverse events”, “immune checkpoint inhibitors”, “immunotherapy”, and “nivolumab” were the most frequently used keywords.

**Conclusions:**

A bibliometric analysis of 17 years of irAEs research was conducted to map a basic knowledge structure including countries, institutions, authors, journals, and publications. The findings provided a comprehensive perspective on the broad future of this research area.

## 1 Introduction

Immune checkpoint inhibitors (ICIs) have emerged as one of the novel and practical approaches to cancer therapy, providing tremendous clinical benefits to patients with cancer. ICIs can damage self-tissue while killing tumors, resulting in a series of toxic side effects that we call immune-related adverse events (irAEs) (1). Due to the prevalence of ICIs, about 54%-76% of cancer patients experience irAEs, including severe toxic reactions (e.g., myocarditis) or permanent toxic reactions (e.g., autoimmune diabetes) (2). Therefore, there is an increasing emphasis on the research, diagnosis, and management of irAEs. However, conducting clinical studies on a large scale is difficult due to the significant heterogeneity of irAEs (3). Even though there are more and more publications on irAEs, a complete analysis of publications, countries, institutions, journals, authors, and keywords is still lacking.

Pritchard introduced bibliometrics in 1969, which was defined as “the application of mathematical and statistical methods to the computation and analysis of different aspects of textual information to reveal the processes of textual information and the nature and trends in the development of a discipline (4).” Currently, bibliometrics is widely used to investigate the characteristics of academic publications (5). For example, it identifies the most influential countries, journals, institutions, and authors in a research field (6). It helps researchers identify high-frequency cited publications and keywords. It also helps visualize and analyze the collaboration between countries, institutions, and authors (7). In addition, bibliometrics can help researchers quickly grasp a specific research field’s evolution and research frontiers. Several bibliometric analyses have investigated the trends and hot topics in the field of immunotherapy (8–10). In the bibliometric analyses targeting immunotherapy in hepatocellular carcinoma and colorectal cancer, both irAEs were found to be an essential topics.

Furthermore, a bibliometric study of 11,971 publications on ICIs from 2000 to 2020 revealed that irAEs formed a unique cluster in the keyword co-occurrence analysis (11). This indicates that irAEs are becoming a widely followed issue in immunotherapy. The bibliometric analysis of irAEs, however, has not been published yet. The purpose of this bibliometric analysis was to fill this gap by creating a global knowledge mapping of scientific publications about irAEs.

## 2 Methods

### 2.1 Data source and publication search strategy

Web of Science (WoS) incorporates over 12,000 journals and is one of the most frequently accessed academic databases (12). When the bibliometric analysis was performed against other databases such as Scopus, Medline, and PubMed, WoS emerged as the most comprehensive and reliable (13). In the present research, the relevant publications were searched and exported to the Web of Science Core Collection database (WoSCC) on September 13, 2022. All versions of WoSCC were used for the study. After consultation with our senior literature search experts and agreement by all authors, the search strategy was set as follows: [TS = (Immune-related side effect OR Immune-related side effects OR Immune-related adverse reaction OR Immune-related adverse reactions OR Immune-related adverse reactions OR Immune-related adverse effect OR Immune-related adverse effects OR Immune-related adverse event OR Immune-related adverse events OR Immune-related toxicity OR Immune-related toxicities)]. The type of publication included regular and review articles. The publication language was restricted to English to facilitate further literature content analysis. For further analysis, relevant publications were extracted and saved in plain.txt format (including complete records and cited references) (14).

### 2.2 Software tools for performing bibliometric analysis in this study

This study used R version 4.0.1 (15), VOSviewer (16), and CiteSpace (17) for bibliometric analysis.

In bibliometrics and scientometrics, the Bibliometrix R package provides tools for quantitative research. In Bibliometrix, authors are extracted from the AU field, including all authors. Keywords are extracted from the DE field, citations from the TC field, and the year of publication from the PY field.

In this study, Bibliometrix version 4.0.0 was used to

1. Count basic bibliometric metrics such as the number of publications and citations,
2. Determine the frequency of keywords/terms,
3. Calculate the frequency of collaboration between countries, and
4. Visualize a three-field plot for keywords plus analysis.

Bibliometric networks can be constructed and visualized using the VOSviewer software tool (18). Based on the software’s embedded clustering algorithm, VOSviewer can construct and visualize co-occurrence networks of important terms extracted from the scientific literature (19). In addition, VOSviewer supports overlaying visual maps to show the network over time. In this study, we primarily utilized co-authorship analysis and co-occurrence analysis. On the one hand, co-authorship networks were constructed to explore the collaborative relationships between authors and their institutions (20). Alternatively, the co-occurrence network shows how the authors’ keywords are related (21).

CiteSpace is a citation visualization and analysis software. Since the structure, patterns, and distribution of scientific knowledge are presented through visualization, the visualization obtained through this method is also called “scientific knowledge mapping (17).” In this study, it was used to identify highly cited references and keywords that experienced high citation bursts during a particular period.

Additionally, an international collaboration between countries was visualized using the online bibliometric website (https://bibliometric.com/). An exponential growth function in Excel was used to analyze the number of publications published per year.

## 3 Results

### 3.1 General analysis of publication status

**Figure 1:**
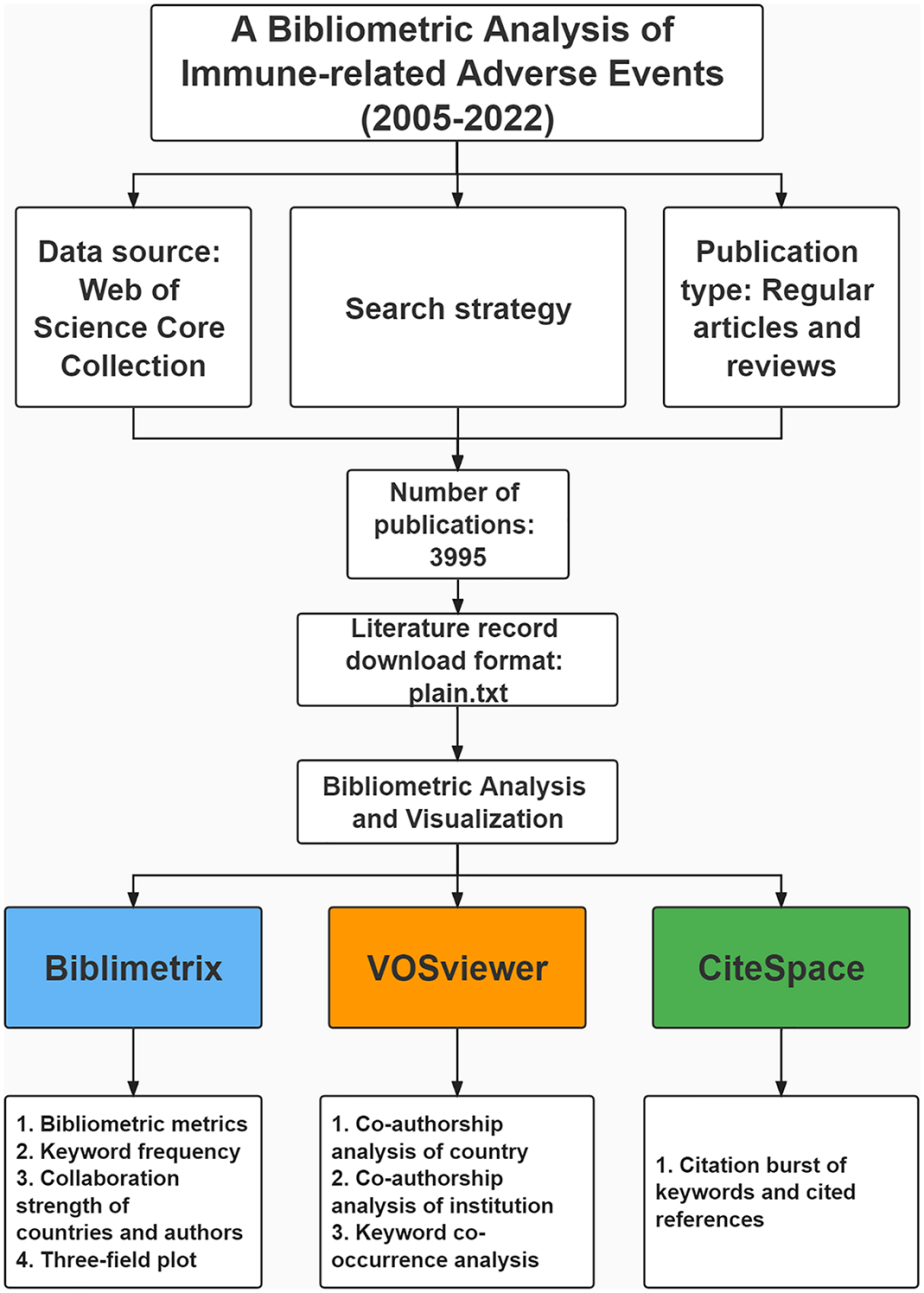
Process and key steps of the study.

**Figure 2:**
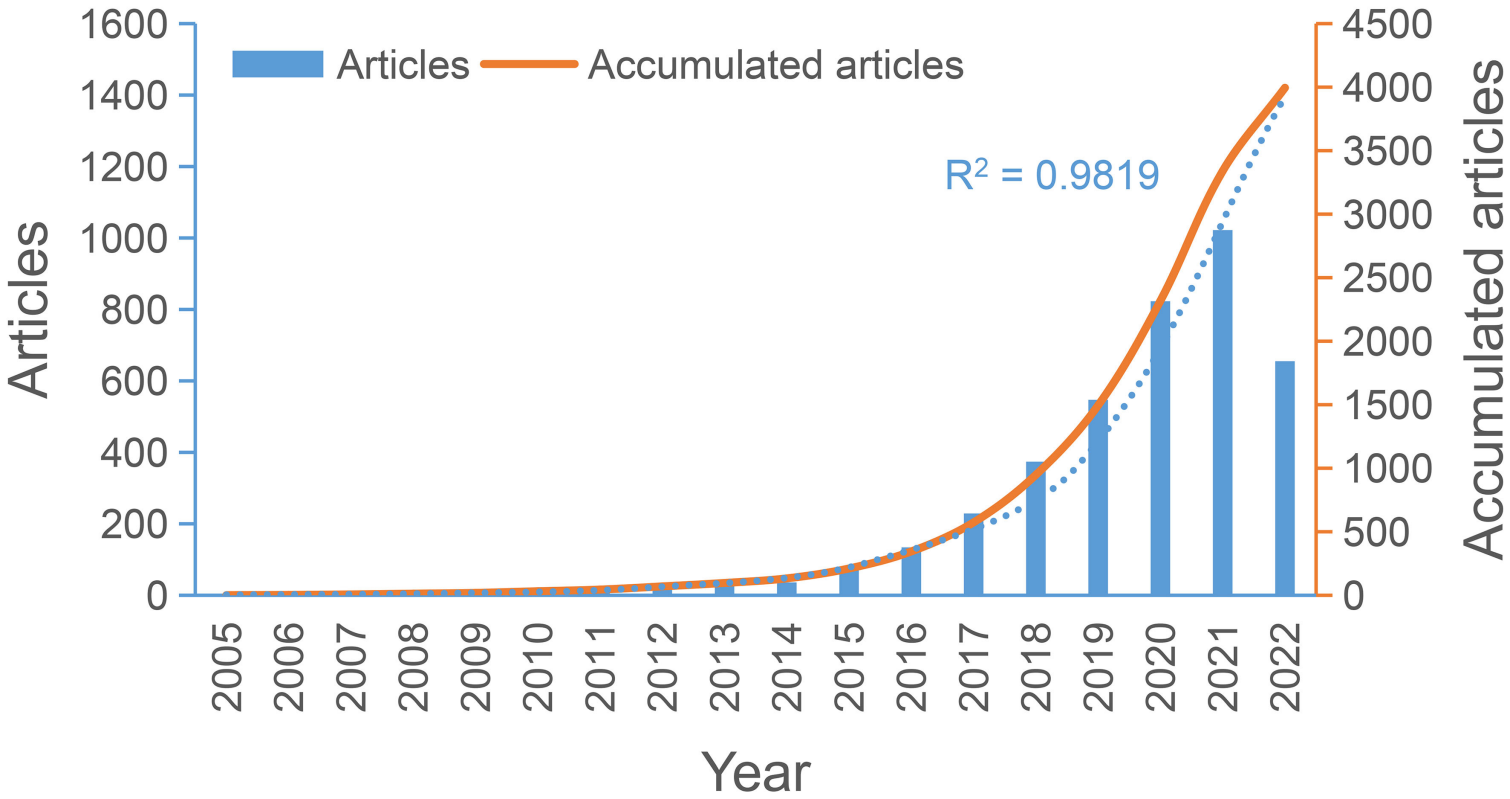
The number of publications per year and the cumulative number.

An overview of the study can be found in **Figure 1**. There were 3995 publications on irAEs, including 2,744 regular and 1251 review papers. **Figure 2** demonstrates the annual number of publications related to irAEs and the cumulative number. A 46.44% annual growth rate was observed. **Supplementary Figure 1** shows the percentage of publication types across years, countries, and authors. Research articles dominate in all dimensions. In 2005, the first article was published in Leukemia. Plumas et al. firsty revealed that mesenchymal stem cells (MSCs) could induce apoptosis in activated T cells, which they indicated could help to develop approaches to control irAEs (22). Overall, the cumulative number of publications steadily increased from 1 in 2005 to 133 in 2014. In the following seven years, the number of publications proliferated until 2021, when the cumulative number of publications reached 3340. In addition, the relationship between the number of publications per year and the year of publication was assessed using an exponential growth model, which matched the trend in the number of publications per year (R^2^ = 0.9819).

### 3.2 Analysis of national publications volume

**Figure 3:**
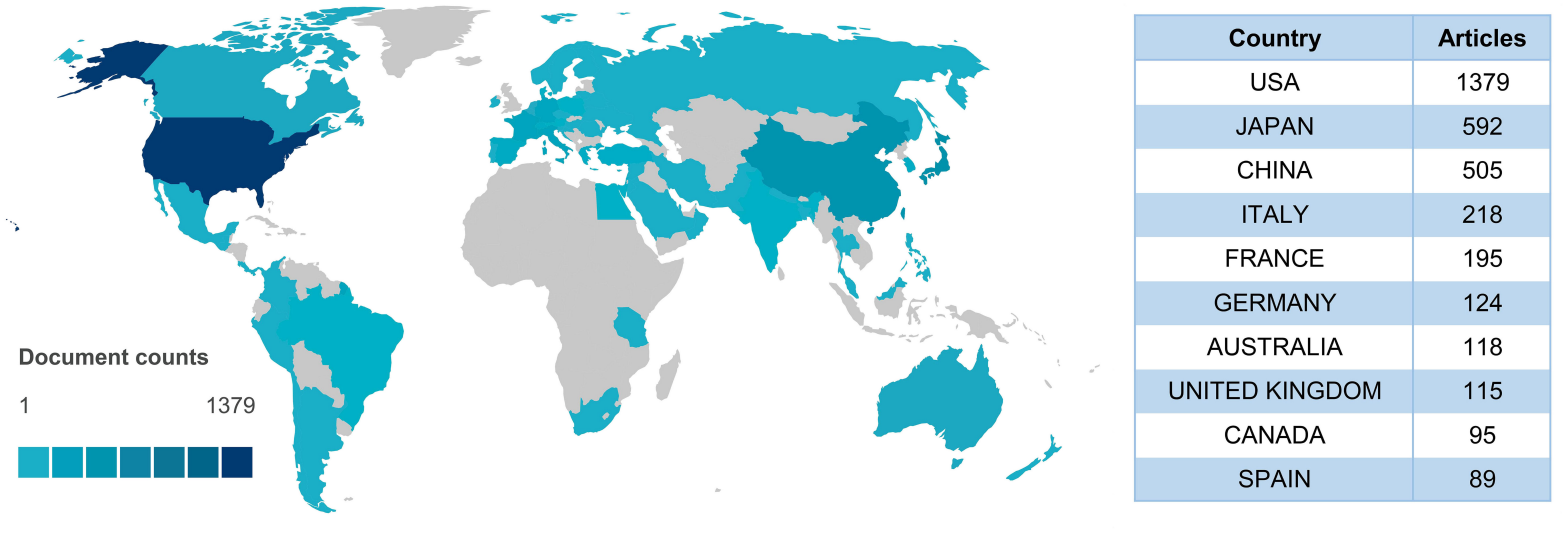
An overview of the contributions of each country based on the number of publications.

In order to explore the countries/regions that contributed the most in the field, an analysis of the number of national publications was conducted. The results are presented in **Figure 3**. The United States (US) ranked first with 1379 publications. It was followed by Japan (592), China (505), and Italy (218). The remaining countries/regions had less than 200 publications.

To further investigate the collaborative relationships between countries/regions, we visualized the country/region collaborations in **Supplementary Figure 2**. The results indicated that the research in the field of irAEs was dominated by the US. The most frequent collaboration was between the US and France (frequency = 75). The following countries were Italy (frequency = 73), the United Kingdom (UK) (frequency = 68), and China (frequency = 67). All of these national collaborators were from the US.

### 3.3 Analysis of institutional publications volume

**Figure 4:**
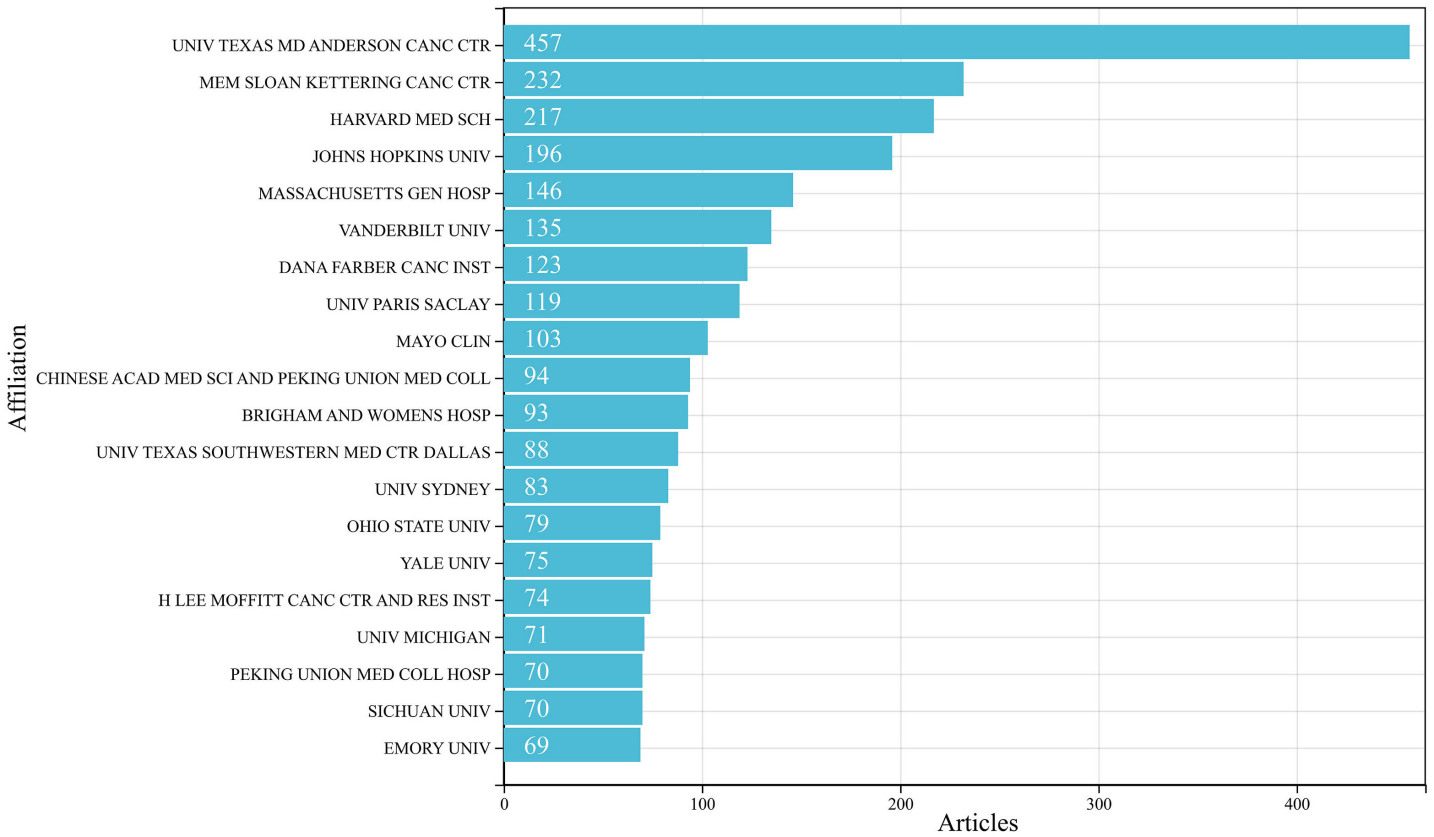
The top 20 institutions with the most publications in the field of irAEs.

To explore the contribution of institutions to the field of irAEs, we analyzed the number of institutional publications. Globally, approximately 4,060 institutions conducted irAEs-related research. The top 20 research institutions are summarized in **Figure 4**. There were 15 US institutions, 3 Chinese institutions, 1 French institution, and 1 Australian institution. The University of Texas MD Anderson Cancer Center ranked first with 457 publications.

A co-authorship analysis was performed on all publications to investigate inter-institutional collaborations further. In the clustering network for the co-authorship analysis and the time-overlapping network, the size of the circles indicate the number of publications. In the clustering network, the color of the circles represented the research groups automatically classified according to the intensity of collaboration. In the time-overlapping network, the circle’s color represented the average year of publication start for each institution in the particular research area. As shown in **Supplementary Figure 3A**, 119 institutions were identified as having published at least 15 articles. One hundred nineteen institutions formed a total of 8 clusters. The red color refers to the cluster containing the most institutions, with 30 institutions belonging to this cluster, most of which were US institutions. In **Supplementary Figure 3B**, research institutions represented by MD Anderson Cancer Institute were early starters in the field of irAEs. In contrast, researchers in China and Japan conducted relatively new research in this area.

### 3.4 An analysis of the number of publications and impact of journals

The 3995 publications included in the research were published in 943 journals. The top 10 journals and their latest impact factors (IF) were listed in **Table 1**, sorted by the number of publications. Five of the top 10 journals were classified in Journal Citation Reports (JCR) Quartile 1 (Q1). Three publishers each from the US, UK, and Switzerland, and one other publisher from Egypt.

**Table 1:** Top 10 journals with most publications in the field of immune-related adverse events.

Rank	Sources	Articles	Country	IF	JCR-c
1	JOURNAL FOR IMMUNOTHERAPY OF CANCER	182	UK	12.469	Q1
2	FRONTIERS IN ONCOLOGY	116	Switzerland	5.738	Q2
3	FRONTIERS IN IMMUNOLOGY	94	Switzerland	8.786	Q1
4	CANCERS	80	Switzerland	6.575	Q1
5	JOURNAL OF IMMUNOTHERAPY	76	US	4.912	Q2
6	EUROPEAN JOURNAL OF CANCER	67	Egypt	10.002	Q1
7	CANCER IMMUNOLOGY IMMUNOTHERAPY	61	US	6.63	Q1
8	ONCOLOGIST	57	US	5.837	Q2
9	IMMUNOTHERAPY	55	UK	4.04	Q3
10	JOURNAL OF ONCOLOGY PHARMACY PRACTICE	52	UK	1.416	Q4

### 3.5 Author influence analysis

A total of 20,734 authors participated in irAEs-related studies. As demonstrated in **Table 2**, Johnson DB was the most productive author with 47 articles and H-index of 22. He was followed by Wolchok JD (40 publications, H-index=30) and Zhang L (37 publications, H-index=9).

**Table 2:** Top 10 authors with the most publications in the field of immune-related adverse events.

Rank	Authors	Articles	H-index
1	JOHNSON DB	47	22
2	WOLCHOK JD	40	30
3	ZHANG L	37	9
4	NAIDOO J	35	21
5	ROBERT C	34	26
6	HODI FS	34	24
7	LAMBOTTE O	32	20
8	REYNOLDS KL	31	14
9	MARABELLE A	29	19
10	MICHOT JM	29	19

**Supplementary Figure 4A** illustrates the clustering diagram of collaborative relationships among researchers. The circle size represents the number of publications, and the color represents the clusters. Seventy-seven authors with several publications greater than or equal to 10 were clustered into 10 clusters. Three clusters were scattered outside of a larger community consisting of 7 clusters. There were no collaborative relationships between the different communities. It suggested that collaboration between research teams/labs conducting research related to irAEs must be further strengthened. **Supplementary Figure 4B** depicts the time-overlapping network of clustering results. We observed that researchers from China, represented by Mengzhao Wang, were forming new research networks on irAEs. Given that collaboration among different research groups are insufficient, national and inter-institutional collaboration is one of the future directions.

### 3.6 Research hotspot analysis

#### 3.6.1 Most cited publications

The frequency of citations in a particular field can indicate research impact; citation counts can be used to assess the most cited articles. **Supplementary Table 1** lists the ten most cited publications between 2010 and 2018, 60% of which have been cited more than 1000 times.

The most cited article was published in 2010 and was titled “Improved Survival with Ipilimumab in Patients with Metastatic Melanoma (23).” The study reported the survival of patients with metastatic melanoma treated with ipilimumab plus gp100 and the probability and severity of irAEs. The authors further pointed out that appropriate treatment could improve most irAEs. The second most cited publication was also published in The New England Journal of Medicine. Postow et al. published a review entitled “Immune-Related Adverse Events Associated with Immune Checkpoint Blockade” in 2018. In this review, the authors focused on ten critical questions about immunotherapy, for example, whether the occurrence of irAEs is related to the effectiveness of treatment with ICIs, providing a valuable reference for researchers to understand irAEs (24).

#### 3.6.2 Reference citation burst analysis

**Supplementary Figure 5** illustrates the burst of the top 20 most cited references. The minimum duration of the burst is two years. The blue line represented the observed time interval from 2005 to 2022, while the red line represented the duration of the burst. The article “Improved Survival with Ipilimumab in Patients with Metastatic Melanoma,” published in The New England Journal of Medicine, had the strongest citation burst value (citation burst = 148.18) between 2011 to 2018 (23). In addition, citation bursts continued for four articles, including “Management of Immune-Related Adverse Events in Patients Treated With Immune Checkpoint Inhibitor Therapy: American Society of Clinical Oncology Clinical Practice Guideline,” which had the highest burst value of 67.11 (25). This article is a practice guideline of the American Society of Clinical Oncology Clinical and has contributed to the management of irAEs. The second most popular article was “Fatal toxic effects associated with immune checkpoint inhibitors: a systematic review and meta-analysis.” Wang et al. reported the incidence and timing of fatal toxic effects associated with ICIs (26). In the future, this type of research topic may remain popular and become a potential frontier in the research on irAEs.

#### 3.6.3 Frequency of keyword occurrence and clustering analysis

**Figure 5:**
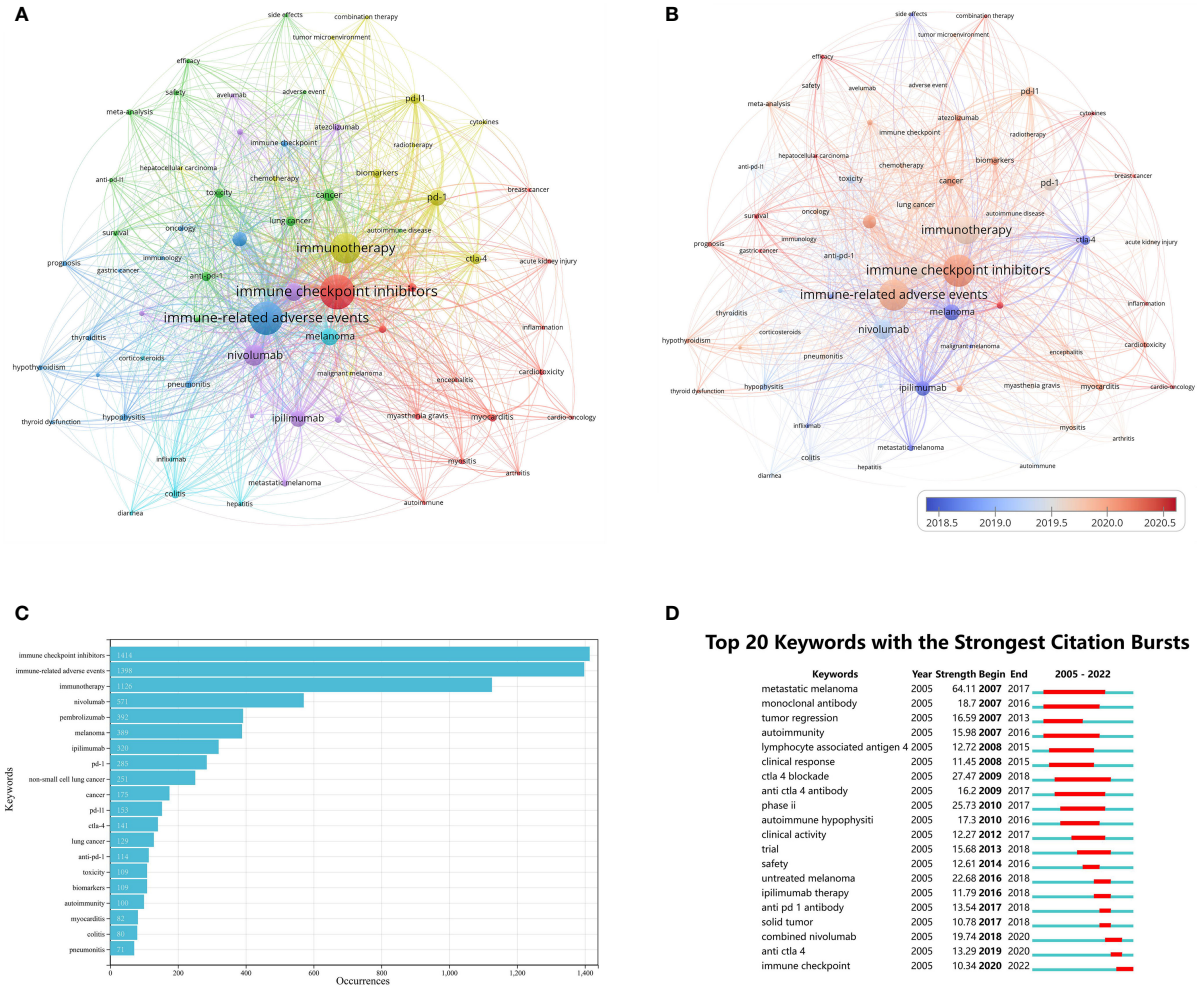
Research hotspots on irAEs **(A)** keyword co-occurrence network of authors; **(B)** time-overlapping co-occurrence analysis network of author keywords; **(C)** a list of the 20 most frequently used keywords; **(D)** the 20 keywords with the strongest citation bursts).

The minimum number of occurrences was set to 20, and 68 of the 4865 keywords met the criteria and were included in the analysis. The keywords were combined if they had similar meanings. The network visualization of these keywords is shown in **Figure 5A**. Node size reflects keyword frequency, while the distance between nodes indicates the strength of their relationship. The 68 keywords were divided into six clusters, reflecting the critical topics in the field of irAEs research. Keywords that are more closely related were assigned to the same cluster. Cluster 1 was red, and the primary keywords focused on the manifestation of irAEs in different systems, such as “acute kidney injury,” “arthritis,” “myocarditis,” and “encephalitis.” Cluster 2 was green and focused on various widely used ICIs with the main keywords “anti-ctla-4”, “anti-pd-1”, “anti-pd-l1” and “efficacy.” In addition, some terms, such as “safety” and “survival,” were included in Cluster 2. Cluster 3 was in blue and concentrated on the description of irAEs with various cancers, with the main keywords being “immune-related adverse events,” “non-small cell lung cancer,” and “oncology.” Cluster 4 in yellow focused on different immune checkpoints and potential biomarkers, mainly involving “pd-1”, “pd-l1”, “ctla-4”, “cytokines,” and “biomarkers.” Cluster 5 in purple mainly comprised a variety of ICIs that have been approved by the Food and Drug Administration (FDA), such as “atezolizumab,” “durvalumab,” “ipilimumab,” and “nivolumab.” The sixth cluster in light blue included primarily “colitis,” “diarrhea,” “hepatitis,” and “infliximab.” It seems to be about irAEs of the digestive system and treatment. **Figure 5B** illustrates the time-overlapping visualization of the authors’ keywords. Earlier appearing keywords were presented in blue, while red indicated recent keywords. Early periods of research focused primarily on “melanoma,” “metastatic melanoma,” “ipilimumab,” and “ctla-4.” In contrast, recent research has focused on the topics of “combination therapy,” “efficacy,” “hepatocellular carcinoma,” “gastric cancer,” “tumor microenvironment,” and “cytokines.”

**Figure 6:**
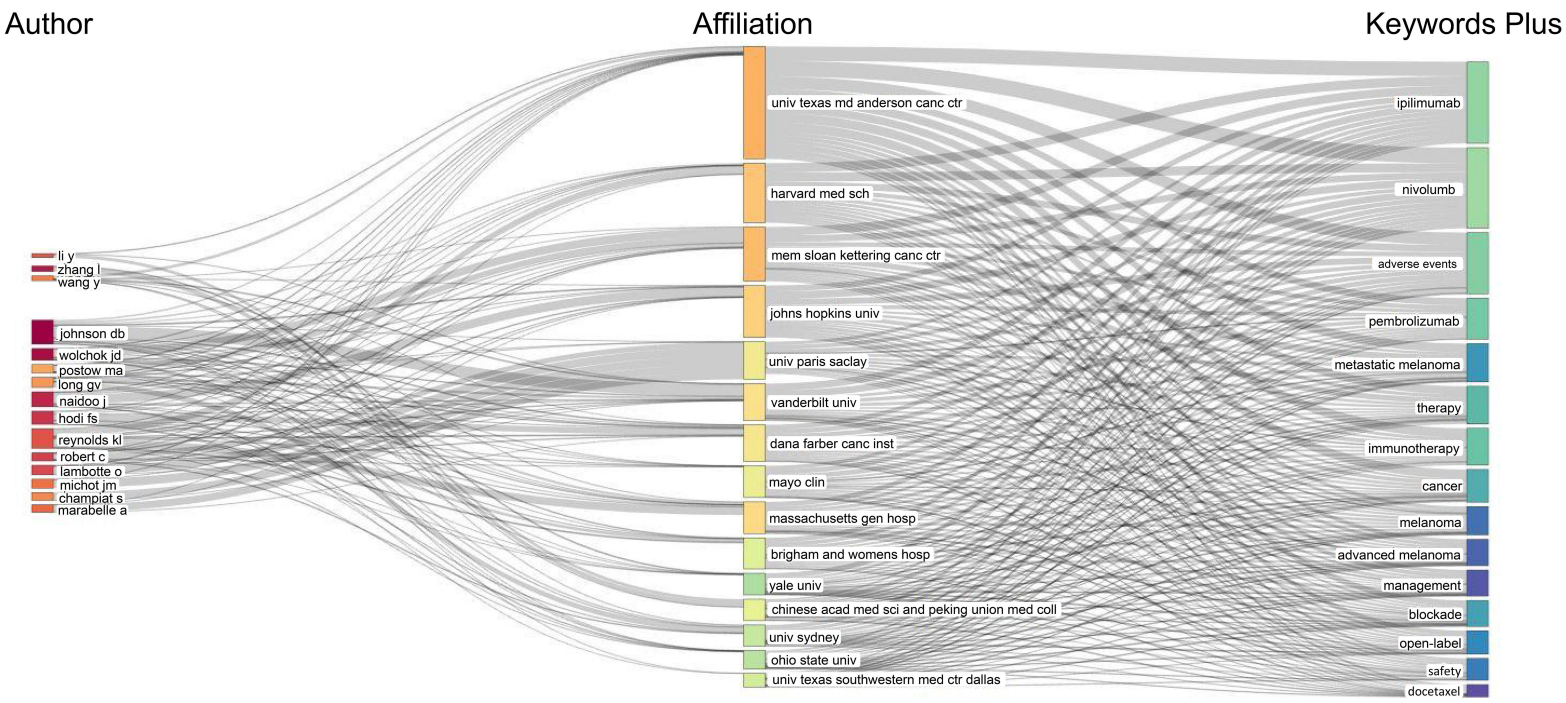
Three-field plot of the keywords plus analysis on irAEs (left field: authors; middle field: affiliations; right field: keywords plus).

**Figure 5C** shows the top 20 keywords in order of frequency of occurrence, where “ immune checkpoint inhibitors” was the most frequently used keyword with 1414 occurrences, followed by “immune-related adverse events” (N = 1398) and “immunotherapy” (N = 1126). Among the top 20 keywords, “non-small cell lung cancer” (N = 251) and “lung cancer” (N = 129) were the only cancer types that appeared. **Figure 6** further demonstrates the association between authors, institutions, and keywords in the field of irAEs research.

#### 3.6.4 Keywords citation burst analysis

In **Figure 5D**, we present the top 20 keywords with the most robust citation bursts, with a minimum duration of one year. The keywords “metastatic melanoma” (2007–2017), “monoclonal antibody” (2007–2016), and “autoimmunity” have received the most protracted attention over time. While keywords such as “combined nivolumab” (2019–2020), “anti ctla 4” (2019–2020), and “immune checkpoint” (2020–2022) have been used more recently, indicating that these keywords have attracted enough attention to become popular research topics in the future.

## 4 Discussion

The present research analyzed the growth pattern of irAEs-related studies from 2005 to 2022 using a bibliometric approach. The growth trend of irAEs-related research could be divided into 2 phases according to whether the annual publications exceeded fifty. Before 2015 was a slow growth phase with less than 50 publications per year. From 2015 onwards, irAEs-related studies entered a rapid growth phase, with the annual number of publications exceeding more than fifty each year. Until 2021, the annual publication volume reached 1022 publications. It indicates that irAEs-related research has started to enter a rapid development stage. The potential reason might be that with the widespread ICIs in oncology treatment, the incidence of irAEs increased, and people began to realize that poor irAEs control might affect patients’ benefits (27). As a result, research institutions have been increasing their support for research related to irAEs, and research funding has been increasing, contributing to the high growth rate of the field.

For this research, the top 10 countries published 3430 articles, accounting for 85.9% of the total publications. Developed countries, represented by the US and Japan, dominate these ten countries. China is the only developing country. In addition, the US also dominated international collaborations, with US-centered international collaborations occupying eight positions among the top 10 countries in terms of frequency of collaboration. The above findings further confirmed the vital contribution and leadership of the US in the field of irAEs research. It could be related to the favorable national economic situation of the US with high investment in health care. Extensive international collaborations will be beneficial to the development of the field and the improvement of the overall research level.

Similar to the national distribution of the number of publications, fifteen of the top twenty institutions were in the US. Although ranked 3rd in terms of the number of publications, China had only three institutions in the top 20 of the list. Japan, which ranked 2nd in terms of the number of publications, had no institution in the top 20. In contrast, a French institution ranked 7th with 119 publications. Most of these studies were based on international collaborations, suggesting that seeking extensive collaboration among institutions might be essential to improve research competitiveness when economic or resources are limited.

Peer-reviewed journals are an essential carrier of scholarly publications. Core journals often bear the task of publishing necessary research in the field (28). By analyzing the number of journal publications, we could identify the top journals in the field of irAEs and provide researchers with potential journals to submit. In the field of irAEs research, the top 10 journals have several publications greater than 50. Among them, The Journal for ImmunoTherapy of Cancer has the highest number of publications, with 182 publications. The most significant publications were Frontiers in Oncology (116) and Frontiers in Immunology (94). The impact factor and JCR were essential indicators to evaluate the impact of journals (29). JCR quadratically divided all journals into zones 1,2,3,4 based on the impact factor (30). Among the top 10 journals in terms of the number of publications, Q1 journals account for 50%. Furthermore, although Japan and China contributed significantly to irAEs research, there was a lack of Asian publishers among the top 10 ranked journals. It suggests a need to establish and develop journals with international influence in Asia.

Research hotspots represented scientific topics widely followed by researchers in a specific period and were one of the questions this study tried to answer. The number of citations could be one of the indicators of how influential a scholarly publication was (31). Highly cited publications tend to represent essential topics in the study field. By calculating the number of citations, we could identify the highly cited publications and thus identify research hotspots. The ten most cited publications identified in this study were published from 2010 to 2018 and focused on the clinical manifestations of irAEs and how to manage irAEs effectively. Of the top 10 cited publications, the earliest two were published in 2010.

Interestingly, both studies were about the application of ipilimumab in the treatment of melanoma. Stephen Hodi et al. demonstrated in a phase 3 clinical trial that ipilimumab improved overall survival in patients with previously treated metastatic melanoma (23). In this study, the incidence of grade 3 or 4 irAEs was 10-15%. Wolchok D et al. explored the optimal dose of ipilimumab alone for advanced melanoma in another phase 2 clinical trial. In this study, the authors reported that the incidence and severity of irAEs increased with increasing doses of ipilimumab. The most common grade 3-4 irAEs were gastrointestinal (32). These two critical studies initiated the application of ipilimumab to treat metastatic melanoma.

Subsequently, Weber et al. published a review in 2012 that systematically described the symptoms of irAEs caused by ipilimumab and management strategies (33). It provided an essential reference for oncologists. In 2014, after the first CTLA-4 monoclonal antibody was approved by the US FDA (34), the PD-1 monoclonal antibody was again approved by the FDA for the treatment of metastatic melanoma (35). Between 2015 and 2017, three reviews on immune checkpoint blockade were published, extensively describing the clinical presentation and management of irAEs associated with immune checkpoint blockade (CTLA-4, PD-1, PD-L1) (36–38). The two cases of fulminant myocarditis reported by Johnson et al. also drew widespread attention from researchers on lethal irAEs (39). In addition, Gibney et al. published a review entitled “Predictive biomarkers for checkpoint inhibitor-based immunotherapy” in 2016, highlighting the value of predictive biomarkers in improving the efficacy of immune checkpoint inhibitor therapy (40). Researchers conducted extensive studies on this topic and identified promising biomarkers in various cancer types.

With the widespread use of ICIs, understanding irAEs has gradually improved. Recently, the American Society of Clinical Oncology published a clinical practice guideline for irAEs, which further standardized the management of irAEs (25). Based on sufficient experience in clinical practice, Postow et al. pointed out that the key to improving the treatment of irAEs lies in elucidating the mechanisms of occurrence to develop more effective treatments. Furthermore, the authors raised a critical issue in this review, namely whether the occurrence of irAEs was correlated with the efficacy of ICIs (24). Patients with irAEs have been found to have higher response rates and better outcomes than patients without such events (41). Although these findings require universal verification, the rational management of irAEs for optimal efficacy is a goal that investigators should strive to achieve. We are pleased to note the results of the research by Kleef et al. They attempted to treat advanced cancer patients with a combination of low-dose ICIs (42). According to the study’s results, low-dose ipilimumab (0.3 mg/kg) plus nivolumab (0.5 mg/kg) had a better irAE profile than the regular regimen without compromising efficacy. As Bakacs et al. suggest, modest activation of the immune system by low doses of ICIs may achieve comparable efficacy with less irAE risk (43). As keywords reflected the core content of the research, co-occurrence analysis could identify high-frequency keywords that appeared simultaneously in different studies. These keywords usually represented the focus of the research field. This study’s most frequently used keywords were “immune checkpoint inhibitors” and “immune-related adverse events.” In addition, other keywords focused on the use of “immune checkpoint inhibitors” in different cancer types and organ-specific irAEs. In addition, “biomarkers” was another frequently occurring keyword. Several studies reported the role of biomarkers in predicting the efficacy of ICIs treatment, disease progression, and recurrence patterns (44–46). However, there were few studies on biomarkers related to irAEs. Since severe irAEs might interrupt treatment, combining approaches to explore biomarkers related to irAEs could provide a powerful tool to maximize individual treatment efficacy.

Burst detection is a bibliometric analysis method provided by CiteSpace. Its primary function is to identify keywords or cited references that appear to have significant shifts over a specific period. Keywords and cited references with burst characteristics imply that they have been widely followed and discussed. It can provide a reference for researchers to explore research hotspots. In this study, “immune checkpoint” was a keyword with a continuous burst since 2020. In addition, there were four cited references in 2020, and the burst has continued. Three of these reviews focused on the management of irAEs (24–26). One study reported five-year survival and the frequency and intensity of adverse events for the combination of nivolumab and ipilimumab in advanced melanoma (47). This is consistent with the development of immune checkpoints and their inhibitors. In light of the above burst detection results, the pathogenetic characteristics of ICIs-associated irAEs and management strategies may be a research direction of interest in the coming period.

We acknowledge that this study has some modest limitations. First, only articles and reviews written in English and recorded in the WoSCC database were included in this study. While this approach may have overlooked some valuable studies, given that WoSCC is the most commonly used database for scientometric analysis and covers the vast majority of studies, we do not believe this will substantially impact overall trends. Second, due to the delay in citation volume, recently published high-quality studies may not have received the attention they deserve and will need to be updated accordingly in subsequent studies. Nevertheless, this study will significantly help relevant researchers to understand the developments, hot spots, trends, and frontiers of irAEs and to identify areas where further research is still needed.

## 5 Conclusion

In conclusion, research on irAEs related to ICIs has received growing attention. The significant increase in annual publications indicates this research area’s growing importance, with the most significant number of publications in the US. The study identified the top researchers and institutions involved in irAE research worldwide. The Journal for ImmunoTherapy of Cancer is the most active in this research field. Wolchok is the most influential author. The pathogenetic characteristics of irAEs and strategies for management were considered hot topics, and the molecular mechanisms by which irAEs occur may be a key direction for future research. As a result, new researchers and policymakers are provided with a comprehensive overview of the field’s evolution and frontiers.

## Data availability statement

The original contributions presented in the study are included in the article/**Supplementary Material**. Further inquiries can be directed to the corresponding author.

## Author contributions

XL, Y-YX, and X-TS conceived and designed the study. S-TJ wrote the manuscript and participated in the design of the study. S-TJ and Y-GL were responsible for analysis and network construction. LZ performed the data analysis and data interpretation. All authors contributed to the article and approved the submitted version.
