# Supplementary material for: Immune-related adverse events: A bibliometric analysis
Source: Front Immunol. 2022 Dec 15;13:1096806. doi: 10.3389/fimmu.2022.1096806 (PMC9797501; doi:10.3389/fimmu.2022.1096806)
Supplement: Supplementary file 2 [file DataSheet_2.docx]

Supplementary Material



**Figure S1.** Percent of articles and reviews (A. percent of articles and reviews published in different years; B. percent of articles and reviews published in the top 10 countries in terms of the number of publications; C. percent of articles and reviews published by the top 10 authors in terms of the number of publications).


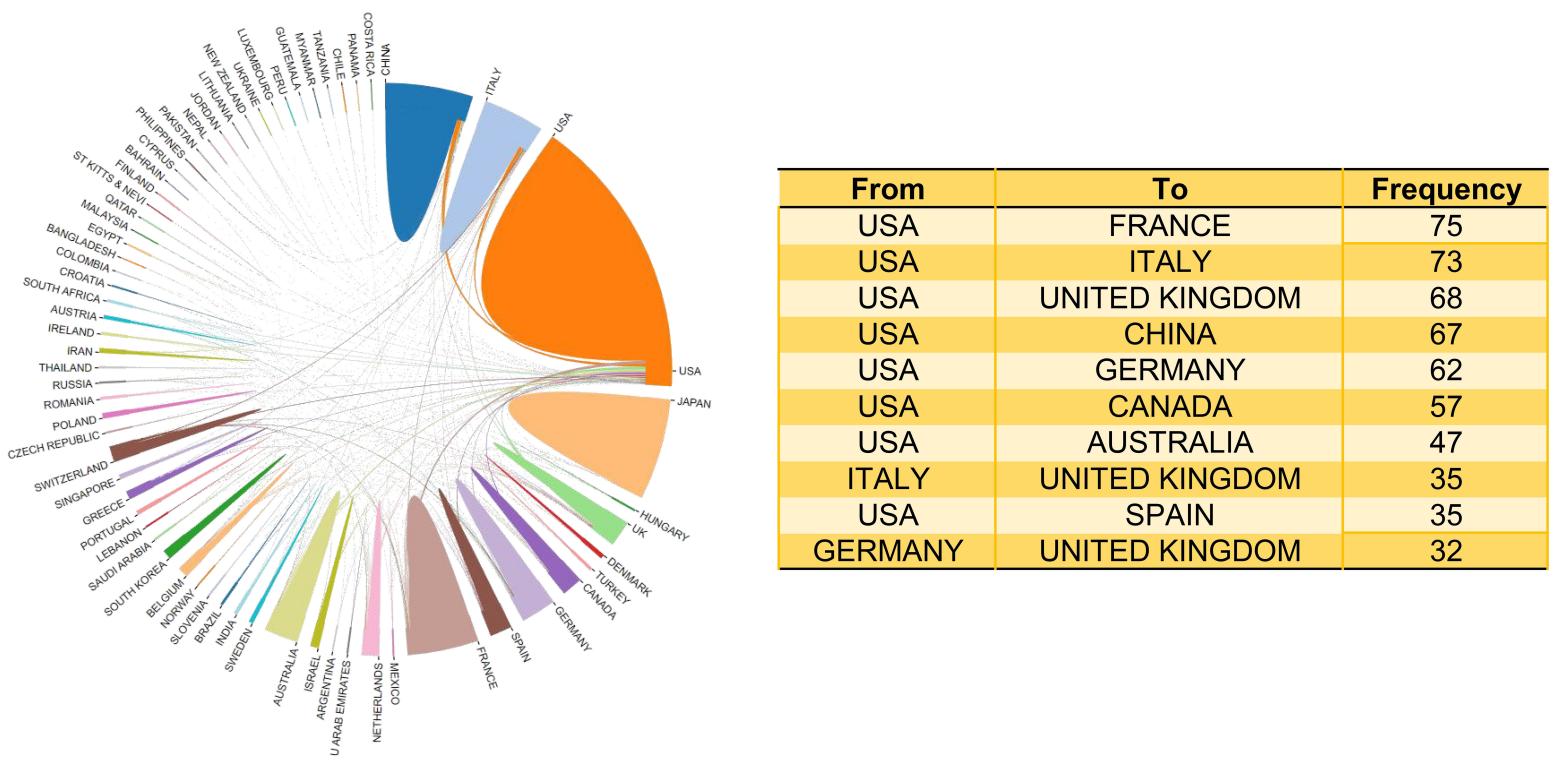


**Figure S2.** International collaboration and high frequency collaboration countries in the field of irAEs research.


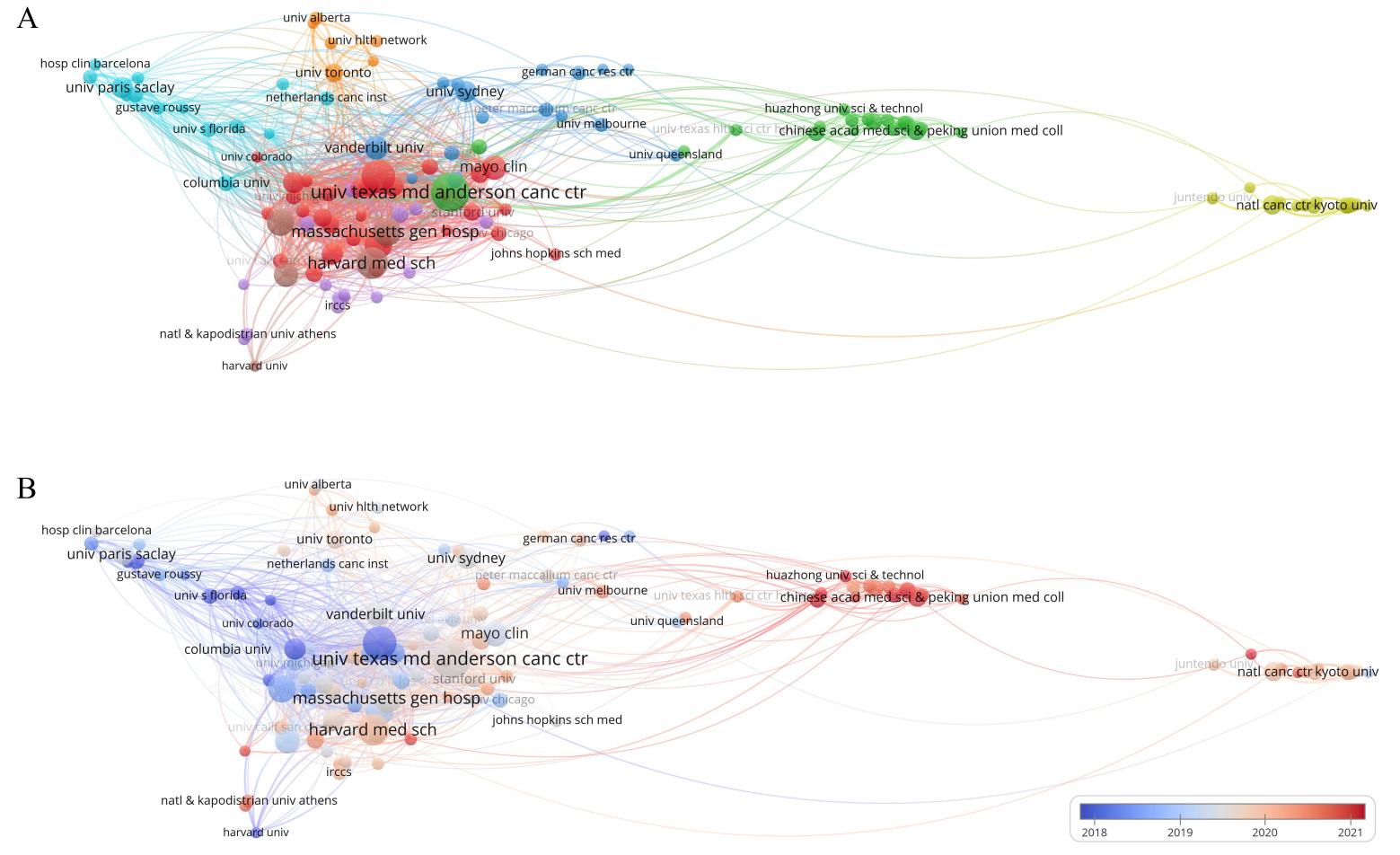


**Figure S3.** Clustering network and time-overlapping visualization mapping for institutional co-authorship analysis.


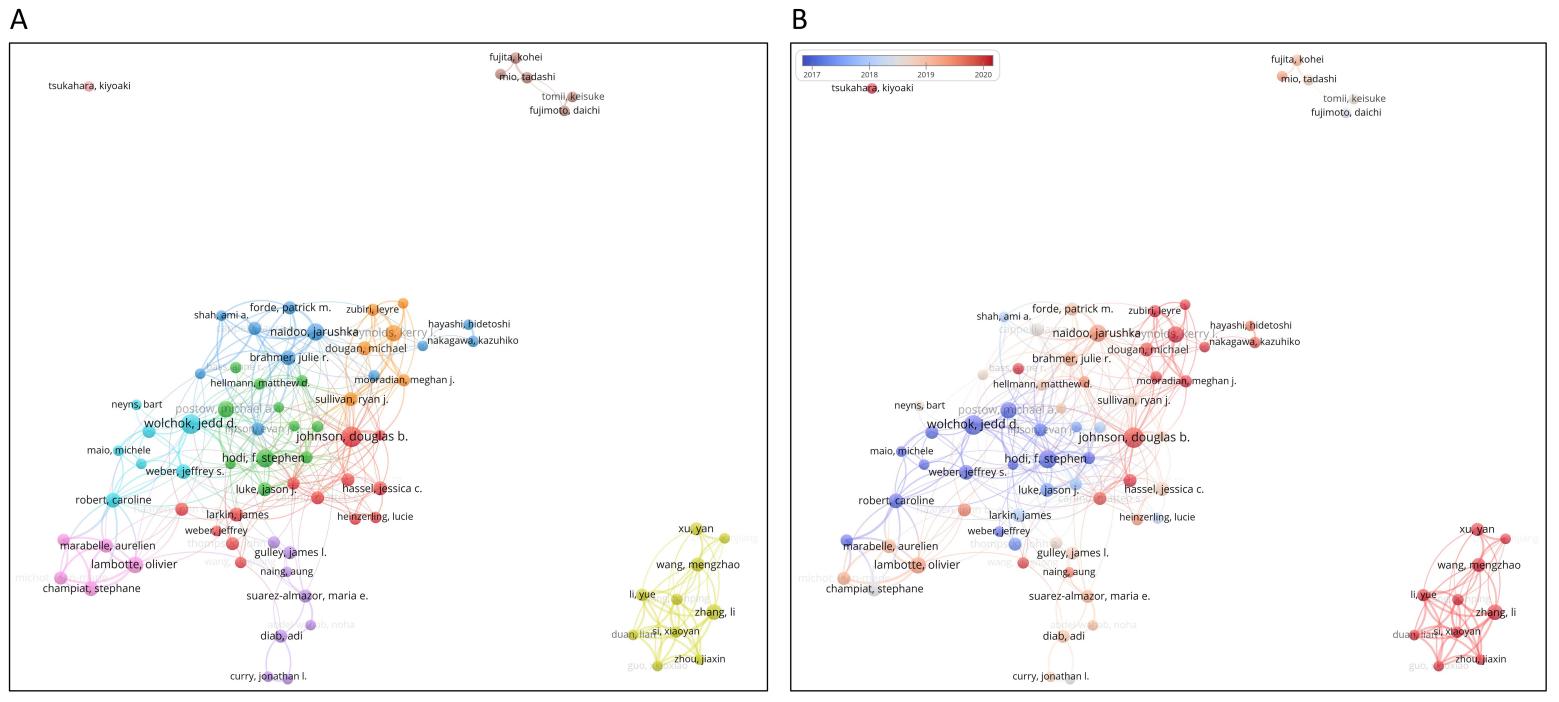


**Figure S4.** Clustering network and time-overlapping visualization mapping for author co-authorship analysis.


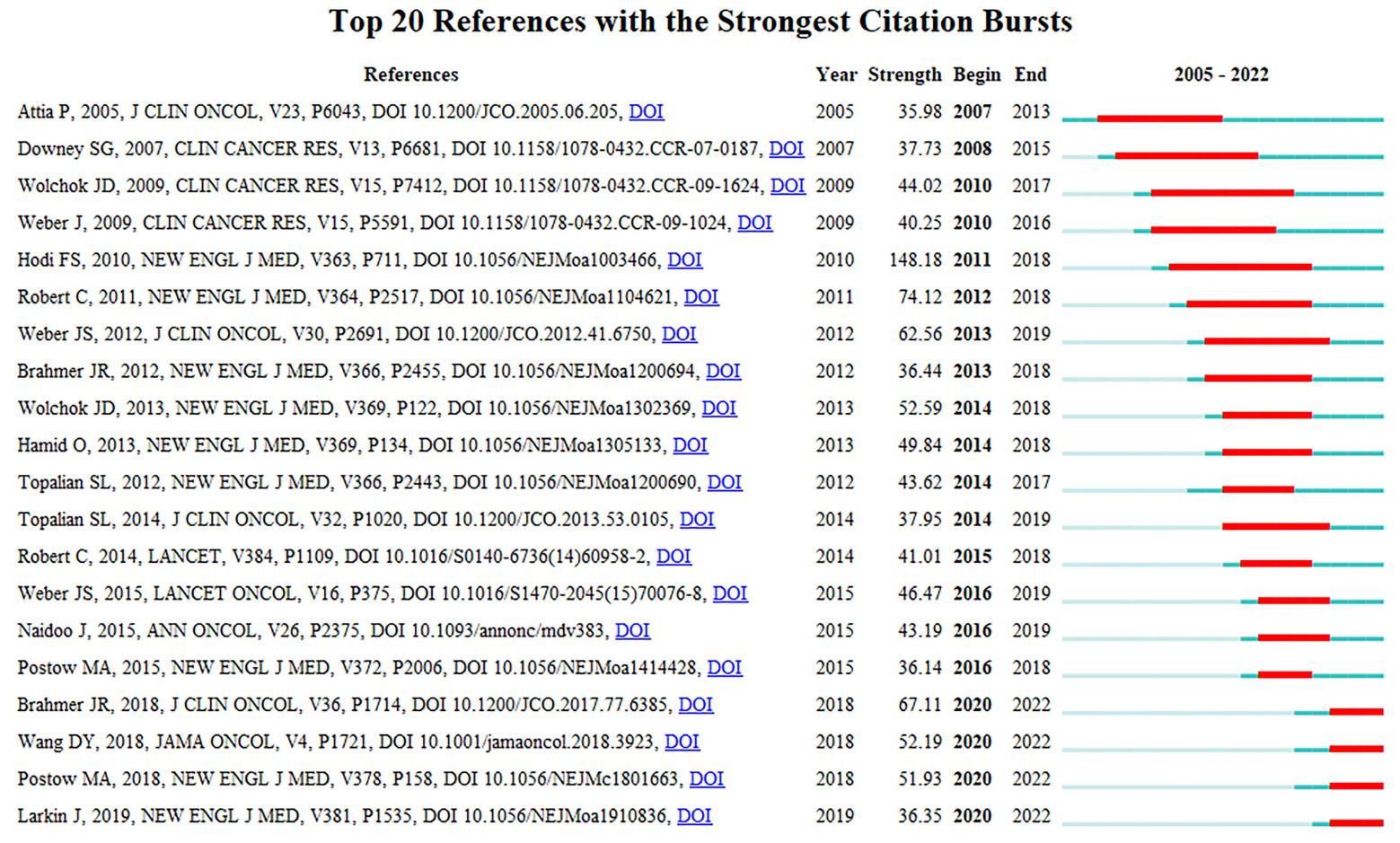


**Figure S5.** Top 20 cited references with the strongest citation bursts on irAEs.

**Table S1.** The top 10 cited publications.

| Rank | Title | Year, Journal | First author | Total citations | TC per Year |
| --- | --- | --- | --- | --- | --- |
| 1 | Improved Survival with Ipilimumab in Patients with Metastatic Melanoma | 2010, NEW ENGL J MED | HODI FS | 10139 | 779.92 |
| 2 | Immune-Related Adverse Events Associated with Immune Checkpoint Blockade | 2018, NEW ENGL J MED | POSTOW MA | 1832 | 366.40 |
| 3 | Management of Immune-Related Adverse Events in Patients Treated With Immune Checkpoint Inhibitor Therapy: American Society of Clinical Oncology Clinical Practice Guideline | 2018, J CLIN ONCOL | BRAHMER JR | 1661 | 332.20 |
| 4 | Immune Checkpoint Blockade in Cancer Therapy | 2015, J CLIN ONCOL | POSTOW MA | 1620 | 202.50 |
| 5 | Immune-related adverse events with immune checkpoint blockade: a comprehensive review | 2016, EUR J CANCER | MICHOT JM | 1201 | 171.57 |
| 6 | Fulminant Myocarditis with Combination Immune Checkpoint Blockade | 2016, NEW ENGL J MED | JOHNSON DB | 1059 | 151.29 |
| 7 | Management of Immune-Related Adverse Events and Kinetics of Response With Ipilimumab | 2012, J CLIN ONCOL | WEBER JS | 987 | 89.73 |
| 8 | Managing toxicities associated with immune checkpoint inhibitors: consensus recommendations from the Society for Immunotherapy of Cancer (SITC) Toxicity Management Working Group | 2017, J IMMUNOTHER CANCER | PUZANOV I | 960 | 160.00 |
| 9 | Predictive biomarkers for checkpoint inhibitor-based immunotherapy | 2016, LANCET ONCOL | GIBNEY GT | 869 | 124.14 |
| 10 | Ipilimumab monotherapy in patients with pretreated advanced melanoma: a randomised, double-blind, multicentre, phase 2, dose-ranging study | 2010, LANCET ONCOL | WOLCHOK JD | 860 | 66.15 |
